# Construction of 1D Ag-AgBr/AlOOH Plasmonic Photocatalyst for Degradation of Tetracycline Hydrochloride

**DOI:** 10.3389/fchem.2020.00117

**Published:** 2020-03-05

**Authors:** Siyang Zhang, Iltaf Khan, Xiaohong Qin, Kezhen Qi, Ying Liu, Shuchong Bai

**Affiliations:** ^1^Institute of Catalysis for Energy and Environment, College of Chemistry and Chemical Engineering, Shenyang Normal University, Shenyang, China; ^2^Key Laboratory of Advanced Energy Materials Chemistry (Ministry of Education), College of Chemistry, Nankai University, Tianjin, China; ^3^Key Laboratory of Functional Inorganic Materials Chemistry, School of Chemistry and Materials Science, Heilongjiang University, Harbin, China; ^4^Department of Information and Control Engineering, Shenyang Institute of Science and Technology, Shenyang, China; ^5^Institute of Paleontological, Shenyang Normal University, Shenyang, China

**Keywords:** Ag-AgBr/AlOOH, plasmonic photocatalyst, methylene blue, tetracycline hydrochloride, degradation

## Abstract

In this work, the highly efficient and low-cost Ag-AgBr/AlOOH plasmonic photocatalyst is successfully prepared via a simple and mild wet-chemical process and used for degrading high concentration methylene blue (MB) and tetracycline hydrochloride (TCH). The optimized 6-Ag-AgBr/AlOOH sample showed a 79% decomposition of TCH in 2 h, which is almost two times higher than that of bare AgBr (37%). For degrading MB, the photocatalytic activity of 6-Ag-AgBr/AlOOH (decomposing 84% in 2 h) showed a large enhancement as compared to bare AgBr (only 57%). The TEM, HRTEM, XRD, DRS, and XPS characterization results confirm that Ag-AgBr is a composite catalyst formed by loading Ag nanoparticles onto AgBr surfaces and then loaded on to AlOOH. The possible mechanism proposed is that •${\text{O}}_{2}^{-}$ and •OH radicals produced under sun light are the main active species for degrading MB and TCH. It is hoped that this work will open a new gateway to the synthesis of highly efficient and low-cost Ag-AgBr/AlOOH plasmonic photocatalysts for degrading organic pollutants.

**Graphical Abstract:**
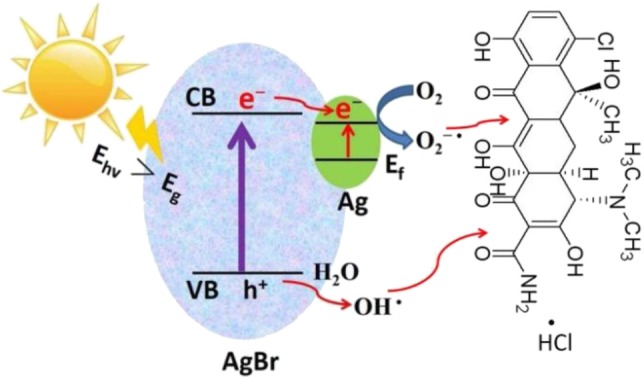
Ag-AgBr/AlOOH photocatalyst is prepared via a simple process and used for degradation of methylene blue and tetracycline hydrochloride.

## Highlights

- Ag-AgBr/AlOOH plasmonic photocatalyst is synthesized.
- Ag and AgBr incorporate on AlOOH supports.
- The •${\text{O}}_{2}^{-}$, •OH radicals are the main active species for degrading TCH.

## Introduction

In recent years, modern industries including the textile, pharmaceutical, paper, plastics, rubber, cosmetics, food, and leather industries use a huge amount of dyes for various purposes (Hassandoost et al., 2019; Hou et al., 2019; Li et al., 2019b, 2020; Qi et al., 2019a; Zhang et al., 2019). These industries discharge liquid waste containing dyes which will affect nature and contaminate drinking water (Li et al., 2019a). For example, methylene blue (MB) is an organic cationic dye that has been found in the drinking water system. Antibiotics such as tetracycline hydrochloride (TCH) are widely used as anti-bacterial and anti-microbial medicines in animal husbandry, aquiculture and the food industry. However, the excessive usage of TCH has created many critical problems, leading to TCH being considered as an influential pollutant (Heidari et al., 2018). To date, many efforts like adsorption, coagulation, chemical oxidation, deposition, membrane separation, biological, physicochemical, and electrochemical techniques have been utilized to remove these dyes and anti-biotics, with a particular focus on MB and TCH (Zhang et al., 2018; Gholami et al., 2019, 2020; Huo et al., 2019b; Kohtani et al., 2019; Li et al., 2019c; Liu et al., 2019b; Wang et al., 2019a). However, long handling times, secondary pollutants and low efficiencies limits the usage of these techniques (Wang et al., 2014; Li et al., 2017, 2018; Karimi et al., 2019; Liu et al., 2019a; Qi et al., 2019b,c; Shen et al., 2019; Takeda et al., 2019).

In the modern era, water contamination has become one of the most critical and common issues for many societies. The above stated traditional, biological and conventional techniques do not fulfill the standard requirements for pollution-free water bodies. Nanotechnology with semiconductor photocatalysis has been considered as an alternative, environmentally friendly and highly efficient method owing to the utilization of sunlight and the lack of secondary contamination (Dai et al., 2012; Dong et al., 2019; Fu et al., 2019; Hayat et al., 2019; Huo et al., 2019a; Liu et al., 2019c; Qi et al., 2019d, 2020b). So far various photocatalysts, including TiO_2_, ZnO, CdS, WO_3_, AgCl, AgBr, etc., have been used, but the poor utilization of solar light has limited the technique's practical utilization (An et al., 2010; Jiang et al., 2012; Qi et al., 2017; Ahmed et al., 2018; Bazazi et al., 2018; Hu et al., 2019; Huo et al., 2019b; Low et al., 2019; Ma et al., 2019; Stucchi et al., 2019; Wang et al., 2019b; Mei et al., 2020). Among these photocatalysts, AgBr has received more and more attention because of its high oxidation efficiency. When the energy of sunlight is greater than the band gap of AgBr, electrons are excited from the valence band (VB) to the conduction band (CB), leaving holes in the VB. Photogenerated electrons and holes are then transferred to the catalyst surface and form highly reactive oxygen species (ROS), such as the superoxide anion (•${\text{O}}_{2}^{-}$) and the hydroxyl radical (•OH). These reactive oxygen species will degrade organic pollutants. Accordingly, researchers from different communities have paid paramount attention to designing visible light active photocatalysts with high efficiency and recyclability for the degradation of organic pollutants (Marzouqi et al., 2019; Qi et al., 2020a).

Aluminum oxy-hydroxide (AlOOH), commonly known as boehmite, is an industrial raw material mostly used as a drug carrier or catalyst support (Duan et al., 2013). Xu et al. (2015) studied the catalytic removal of formaldehyde over Pt deposited AlOOH nanoflakes and confirmed that AlOOH performed exceptionally well. Yan et al. (2016) investigated the enhanced oxidation of formaldehyde by utilizing a Pt supported CeO_2_/AlOOH composite. Building on this idea, AlOOH in this work was synthesized through a simple and mild wet-chemical process, followed by further modification through loading with nanoparticles of Ag and AgBr. It is well-known that noble metal containing nanoparticles/materials have active UV-Vis absorption capabilities attributed to the plasmon effect created by the cumulative oscillations of surface electrons with the main components (Ding et al., 2018). To date, various types of plasmonic photocatalysts. particularly Ag/AgCl-based nanostructures have been utilized for the degradation of color dyes and antibiotics (Zhang et al., 2014; Dai et al., 2019). AgBr has good photosensitive properties and exhibits a high photocatalytic activity originating from the plasmon resonance of Ag as present in AgBr (Cheng et al., 2011).

To the best of our knowledge, a very limited number of work studies have declared the synthetization of a Ag-AgBr/AlOOH plasmonic based photocatalyst for degrading MB and TCH. In this research, compared with the pristine AlOOH, the optimized sample 6-Ag-AgBr/AlOOH showed a big improvement for degrading MB and TCH. The TEM, HRTEM, XRD, DRS, and XPS characterization confirmed that AgBr particles are loaded onto AlOOH nanorods, then Ag nanoparticles are loaded onto AgBr/AlOOH composites. The photocatalytic mechanism proposed is that •${\text{O}}_{2}^{-}$ and •OH radicals are the main active species which degrade MB and TCH. The authors hope that this work will open a new pathway to prepare high efficiency and low-cost Ag-AgBr/AlOOH plasmonic photocatalysts that can be used for the degradation of organic pollutants.

## Experimental

All chemicals and reagents used were of analytical grade and used without further purification. Deionized water was used throughout for all types of experiments. Aluminum chloride hexahydrate, barium nitrate, ammonium bicarbonate, and liquid ammonia were used as received from Aladdin company China.

### Preparation

For the preparation of AlOOH, 0.08 mol aluminum chloride hexahydrate, 0.007 mol barium nitrate, 0.01392 mol ammonium bicarbonate, and 0.48 mol liquid ammonia added to a beaker with 100 ml of distilled water. After stirring for 30 min the solution was poured into a Teflon lined steel autoclave. This was properly sealed and placed into an oven at 230°C for 5 h. After the reaction was complete, the autoclave was allowed to cool naturally to room temperature and the product was collected in the form of a precipitate. After being washed several times with ethanol and water ethanol alternatively, the collected sample was dried in an oven at 80°C for 4 h and finally the sample was collected in the form of a fine powder.

In order to obtain a varying amount of Ag-AgBr loaded onto the AlOOH surface to make the Ag-AgBr/AlOOH composite, 0.2 g of as-synthesized AlOOH was dissolved in 60 mL distilled water and named Solution A. Different volumes of a solution of AgNO_3_ (0.1 molL^−1^) (4, 5, 6, 7, and 8 ml) were added drop wise into separate aliquots of Solution A (the products are named as X-Ag-AgBr/AlOOH, X = 4, 5, 6, 7, 8, respectively.). After 30 min continuous stirring, a solution of KBr (0.1 molL^−1^) was added drop wise into Solution A and stirred for another 10 min. When the solution became homogenous, it was stirred for 3 h in the dark. After completing this process, the solution was exposed to high intensity Xenon lamp light for 1 h. Finally, the whole mixture was allowed to cool naturally to room temperature and the product was washed several times with ethanol and water ethanol alternatively. The as-collected samples were dried in oven at 70°C overnight and the final sample was collected in the form of a fine powder.

### Photocatalytic Activity

The photocatalytic activities for the decomposition of MB and TCH were measured at room temperature. The procedure followed for these experiments was as follows: in a typical experimental run an 100 mL quartz was used. Before starting the photochemical reaction, every 80 mL of MB or TCH solution (MB: 10 mg L^−1^ and TCH: 10 mg L^−1^) of the sample was dispersed with 1 g of AlOOH, AgBr, Ag-AgBr and X-Ag-AgBr/AlOOH photocatalyst, and then allowed to stir for 1 h in the dark in order to reach an adsorption-desorption equilibrium. Then the reaction mixtures were irradiated with visible light to induce a photocatalytic reaction by utilizing a 500 W Xenon lamp (Beijing NBeT Technology Co., Ltd., China). After a 2 h reaction of each experimental run, 5 mL of the reacted solution was extracted from the quartz reactor and filtered into a UV cuvette and then measured using a UV-vis spectrometer.

### Characterization

The X-ray diffraction (XRD) patterns were collected on a Bruker D8 Advance Diffractometer (Germany) with Cu-Kα radiation (λ = 1.5418 Å). X-ray photoelectron spectroscopy (XPS) was carried out using an ESCALAB MKII X-ray photo-electron spectrometer using Mg-Kα radiation. Transmission Electron Microscopy (TEM) was performed on a JEM-2010 instrument. Ultraviolet-visible diffuse reflectance spectroscopy (UV-vis DRS) of the samples was performed using a UV-vis spectrophotometer (UV-3600, Shimadzu) with an integrating sphere attachment. An IVIUM V13806 electrochemical workstation was used to take photoelectrochemical measurements.

## Results and Discussion

### XRD

**Figure 1:**
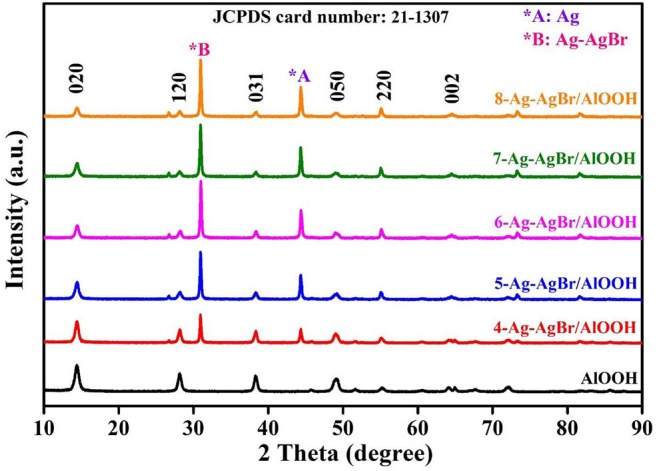
XRD pattern of pristine AlOOH and X-Ag-AgBr/AlOOH composites.

The crystallinity of the samples was investigated by XRD analysis. It can be observed from XRD patterns given in Figure 1 that all of the as-prepared samples possess well-defined diffraction peaks with no impurities, confirming the phase purity of the samples. It can be seen that the XRD patterns are well-indexed to AlOOH (JCPDS card number 21-1307), which matches well with Yan et al.'s results 2016. Moreover, it can be observed from the XRD results that pristine AlOOH has blunt peaks. AgBr and Ag-AgBr have sharp XRD peaks (JCPDS card number 65-2871). When AgBr or Ag-AgBr is loaded onto the AlOOH surface the peak of Ag (44.31°) or AgBr (31.02°) appears accordingly, and the intensity of peak increases as the amount of Ag or AgBr increased. These XRD results confirm that Ag and AgBr are successfully loaded onto the AlOOH surfaces.

### UV-vis-DRS

**Figure 2:**
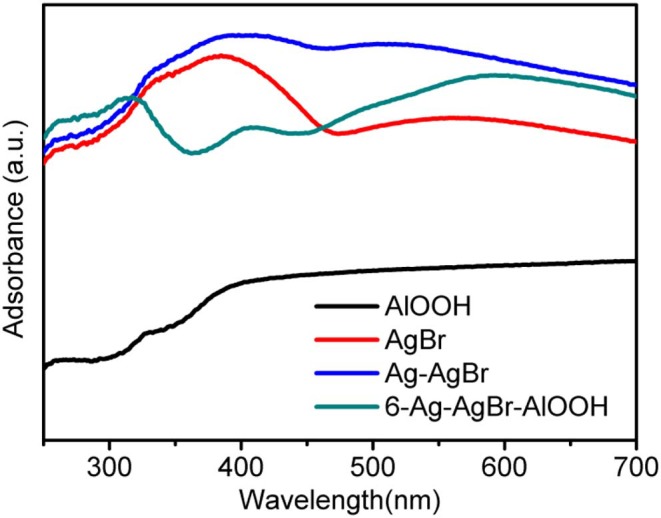
DRS of pristine AlOOH, AgBr, Ag-AgBr, and 6-Ag-AgBr/AlOOH composites.

The DRS spectra of AgBr, Ag-AgBr and 6-Ag-AgBr/AlOOH are given in Figure 2. It can be observed that pure AlOOH shows almost no light adsorption, corresponding to its white color. One can see that the AgBr or Ag-AgBr absorption edge is in a long wave length region (the visible region) at roughly 470 nm. The DRS result of the 6-Ag-AgBr/AlOOH sample shows three characteristic reflection regions: one from 350 to 400 nm, one from 400 to 450 nm, and another at ~600 nm. These reflection regions originate from the metal to metal transition and from the crystal field transitions. The visible light absorption of 6-Ag-AgBr/AlOOH increased with the loading amount of Ag-AgBr, which could be due to the strong surface plasmon resonance (SPR) effect of metallic Ag loaded on the surface of AgBr nanoparticles (Cao et al., 2013).

### XPS

**Figure 3:**
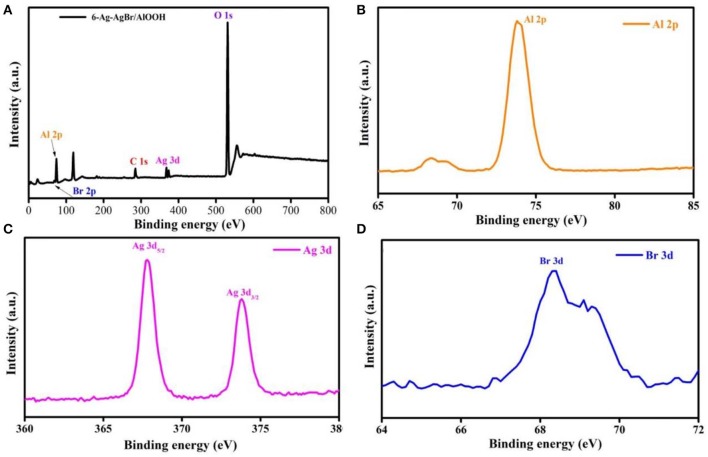
XPS survey spectra **(A)**, Al 2p **(B)**, Ag 3d **(C)**, and Br 3d **(D)** of the 6-Ag-AgBr/AlOOH composite.

XPS is a potential-based technique used to investigate the elemental state and composition of the prepared 6-Ag-AgBr/AlOOH photcatalyst. XPS was carried out to study the effect of loading Ag-AgBr onto the AlOOH surface. Figure 3A shows the total XPS survey of 6-Ag-AgBr/AlOOH composite. It can be noticed that Ag-AgBr was successfully loaded onto the main AlOOH. The XPS peaks show that 6-Ag-AgBr/AlOOH contains only Ag, Br, O, Al, and C atoms. The observed C peak is attributed to the carbon supporting film on the TEM grid. It can be seen more clearly in Figure 3B that the peak at 73.05 eV corresponds to Al 2p (Yan et al., 2016). In Figure 3C the peaks at 367.83 and 373.79 eV are attributed to Ag 3d_5/2_ and Ag 3d_3/2_, respectively (Zhou et al., 2018), whilst the peak at 367.83 eV is assigned to Ag^0^ and the peak at 373.79 eV is attributed to Ag^+^ in AgBr (Zhou et al., 2014). The XPS data of Br 3d is shown in Figure 3D. The peak of the Br 3d binding energy at 66.4 eV is attributed to Br^−^, suggesting the existence of Br atoms in the structure of 6-Ag-AgBr/AlOOH (Zhao et al., 2017). In short, the XPS measurements confirm that Ag and AgBr are loaded onto the AlOOH surface.

### Photocurrent

**Figure 4:**
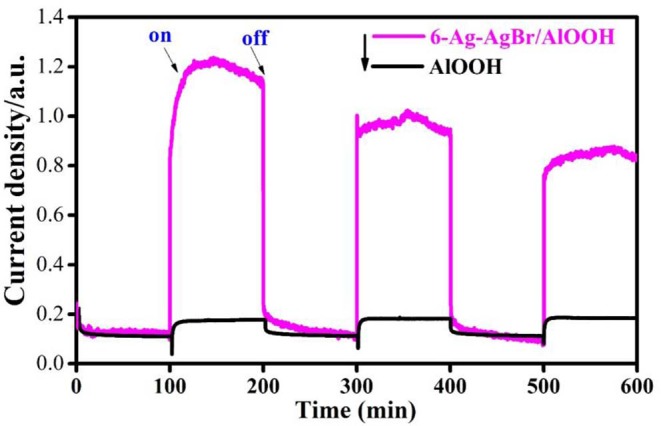
Photocurrents of pristine AlOOH and 6 Ag-AgBr/AlOOH composite.

Photoelectrochemical measurements are considered a key technique to confirm the photoelectro- and electro-chemical nature of samples, so we conducted photoelectrochemical measurements to study the photocurrents of AlOOH and 6-Ag-AgBr-AlOOH samples. The results depicted in Figure 4 confirm that the bare AlOOH has a very weak response when tested using an on/off light. However, for the 6-Ag-AgBr-AlOOH sample, the response to turning light on is significant for up to three on-off cycles. This positive response suggests that the loaded Ag-AgBr particles enhance charge separation, resulting in a reduced charge recombination of the electron (e^−^) and hole (h^+^), and finally enhanced photocatalytic activities for the degradation of organic pollutants.

### TEM

**Figure 5:**
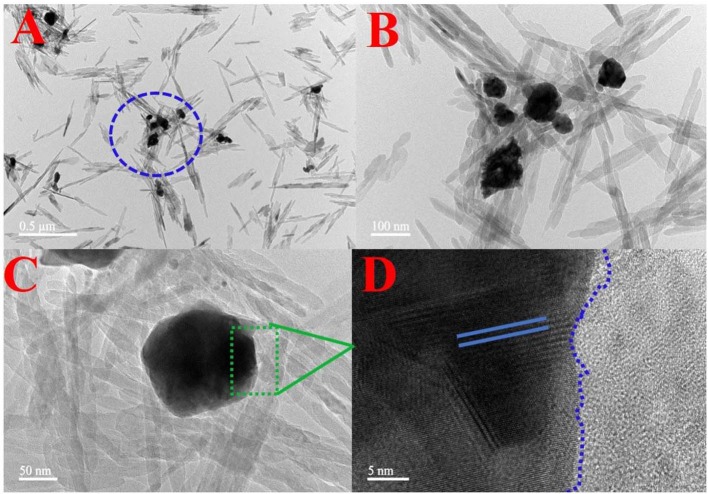
TEM **(A–C)** and HRTEM **(D)** images of the 6-Ag-AgBr/AlOOH composite.

**Figure 6:**
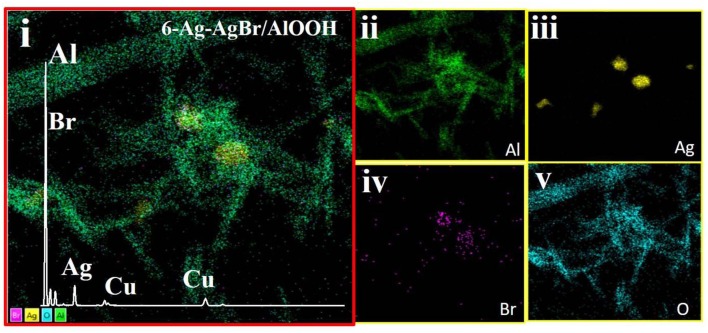
EDS Electron/Survey **(i)** with elemental mapping of Al **(ii)**, Ag **(iii)**, Br **(iv)**, and O **(v)** of the 6-Ag-AgBr/AlOOH composite.

The morphology and elemental map analysis of 6-Ag-AgBr-AlOOH were investigated through TEM measurements. As depicted in Figures 5A–C, the Ag and AgBr are clearly introduced onto the AlOOH surface. The AlOOH sample has a rod-like shape and is about 500 nm long. The size of the Ag-AgBr particles is about 200 nm. It can be seen from HRTEM micrograph (Figure 5D) that different fringes, lines and boundaries are present which confirms that our desired composition procedure has well been accomplished. In order to confirm the existence of the loading materials EDS characterization was carried out, and the results are given in Figures 6i,ii,iii,iv,v. As expected, the Ag and Br are well-distributed onto the surface of the AlOOH nanorods.

### Photocatalytic Activity

**Figure 7:**
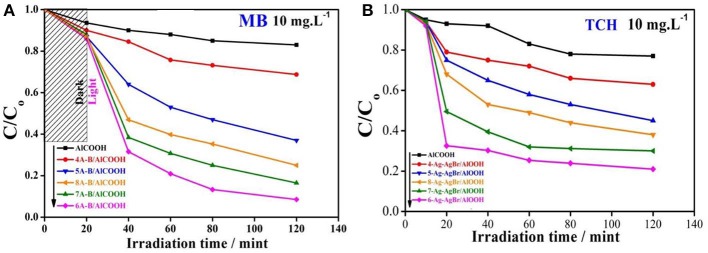
Photocatalytic activity for MB **(A)** and TCH **(B)** of AlOOH and X-Ag-AgBr-AlOOH.

**Figure 8:**
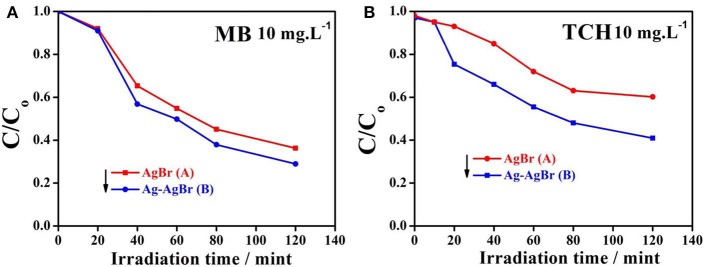
Vis-light photocatalytic activity for MB **(A)** and TCH **(B)** of AgBr and Ag-AgBr.

The photocatalytic activity of pure and Ag-AgBr loaded AlOOH samples was evaluated for degradation of MB or TCH. Figure 7A shows that the AlOOH has a very low photocatalytic activity for the degradation of MB. However, the activity improved after loading with Ag-AgBr, especially for 6-Ag-AgBr-AlOOH which has an activity of 84%, which is 4.9 times greater than that of bare AlOOH (17%). A similar situation can be seen with the photodegradation of TCH as shown in Figure 7B: the activity of 6-Ag-AgBr-AlOOH (79%) is 3.4 times greater in comparison to bare AlOOH (23%). Compared to the data in Figure 8, the optimized 6-Ag-AgBr/AlOOH sample showed a 79% decomposition of TCH in 2 h, which is almost two times greater than that of bare AgBr (37%). For degrading MB, the photocatalytic activity of 6-Ag-AgBr/AlOOH (decomposing 84% in 2 h) showed a large enhancement as compared to bare AgBr (only 57%). When the photocatalytic activity of a AgBr loaded sample is compared with an Ag-AgBr loaded sample, the improvement in activity by loading Ag onto AgBr is attributed to the plasmonic effect of Ag nanoparticles, via the collective oscillations of surface electrons, which promotes the charge separation in AgBr.

### Photocatalytic Mechanism

**Figure 9:**
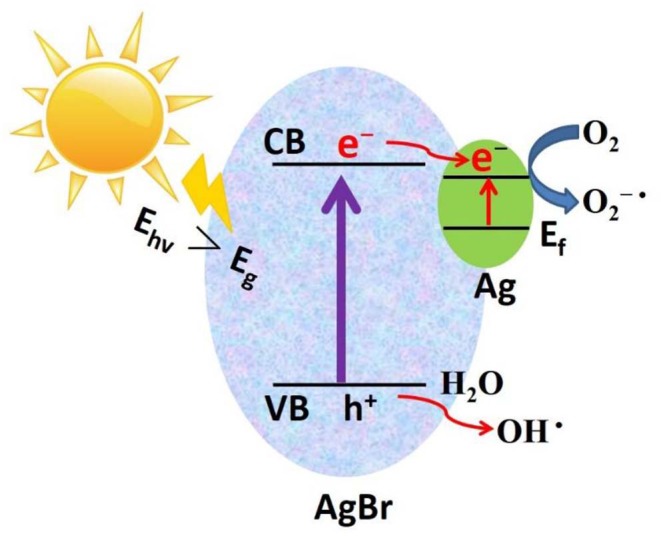
Proposed photocatalytic mechanism of Ag/AgBr/AlOOH composites.

Based on the above experimental results, a possible photocatalytic mechanism was proposed as shown in Figure 9. First, due to the strong electron trapping effect of surface Ag nanoparticles, electrons can be easily transferred from the conduction band of AgBr to the Ag nanoparticles and produce ROS (O^2−·^). After this O^2−·^ can join the reaction to degrade organic pollutants. Secondly, due to the strong surface plasmon resonance (SPR) of Ag nanoparticles, the visible light absorption will enhance, which results in extra enhancement of the photocatalytic activity at the visible light region. In the valence band of AgBr, the photogenerated holes will react with H_2_O to generate ^·^OH radicals. Using AlOOH as a catalyst support not only offers a proper carrier for the catalyst Ag-AgBr, but also dramatically decreases the cost of raw materials.

## Conclusions

In this work, the Ag-AgBr-AlOOH photocatalyst was successfully synthesized via a simple and mild wet-chemical process. The photocatalytic performance of AlOOH (if any) is enhanced by loading different amounts of Ag-AgBr. The as-prepared samples are highly active for degrading MB or TCH under visible light irradiation. The utilization of various characterization techniques confirms that the AgBr and Ag are successfully loaded onto AlOOH surfaces. The collectively loaded AgBr and Ag is a good choice for a promoter because Ag creates a plasmonic effect which not only speeds up charge separation but also improves visible light adsorption, resulting in an enhanced photocatalytic performance. This work will open a new gateway to synthesizing plasmonic nature based photocatalysts for use in environmental purification.

## Data Availability Statement

The datasets generated for this study are available on request to the corresponding author.

## Author Contributions

SZ conducted the catalysts preparation. YL performed the activity test. XQ and IK discussed the mechanism part. SB and KQ conceived the project and co-wrote the manuscript.

### Conflict of Interest

The authors declare that the research was conducted in the absence of any commercial or financial relationships that could be construed as a potential conflict of interest.
